# Detection of Gastroesophageal Reflux Esophagitis Using 2-fluoro-2-deoxy-d-glucose Positron Emission Tomography

**DOI:** 10.1100/2012/702803

**Published:** 2012-07-31

**Authors:** Min-Kuei Tsai, Hueisch-Jy Ding, Hsueh-Chou Lai, Kuo-Yang Yen, Chia-Ing Li, Yu-Yi Lin, Kai-Yuan Cheng, Keh-Bin Wang, Chia-Hung Kao

**Affiliations:** ^1^Department of Medical Imaging and Radiological Sciences, Central Taiwan University of Science and Technology, Taichung 40601, Taiwan; ^2^Department of Nuclear Medicine, Kuang Tien General Hospital, Taichung 433, Taiwan; ^3^Department of Medical Imaging and Radiological Sciences, I-Shou University, Kaohsiung 84001, Taiwan; ^4^Division of Gastroenterology, Department of Internal Medicine, China Medical University Hospital, Taichung 404, Taiwan; ^5^Department of Nuclear Medicine and PET Center, China Medical University Hospital, Taichung 404, Taiwan; ^6^Department of Medical Research, China Medical University Hospital, Taichung 404, Taiwan; ^7^Graduate Institute of Clinical Medicine Science and School of Medicine, College of Medicine, China Medical University, Taichung, Taiwan

## Abstract

*Background*. Gastroesophageal reflux disease (GERD) is a common disease and a major upper gastrointestinal problem. The purpose of the present study is to evaluate the use of noninvasive 2-fluoro-2-deoxy-d-glucose positron emission tomography (FDG-PET) to detect gastroesophageal reflux esophagitis. *Materials and Methods*. This is a retrospective study reviewing 408 healthy check-up subjects (169 females and 239 men), who underwent both FDG-PET and upper gastrointestinal endoscopy during September 2008 to December 2009. Quantitative analysis of FDG uptake in the distal part of the esophagus was performed by calculating the maximum standard uptake value (SUVmax). This indicated the degree of esophagitis. FDG-PET findings were compared with endoscopic (modified version of the Los Angeles classification) diagnoses as the gold standard. *Results*. The SUVmax ranged from 1.30 to 3.40 in normal subjects and from 1.30 to 4.00 in subjects with gastroesophageal reflux esophagitis. In the esophagitis group, the SUVmax was 2.13 ± 0.42 in subjects with modified LA grade M, 2.21 ± 0.45 in subjects with LA grade A, and 2.48 ± 0.44 in subjects with LA grade B and C gastroesophageal reflux esophagitis. One-way ANOVA and post-hoc comparison with Bonferroni correction (*P* value = 0.003) identified statistical differences between the three groups. *Conclusion*. Noninvasive FDG-PET may be useful in the detection and evaluation of various degrees of gastroesophageal reflux esophagitis.

## 1. Introduction

In Asia, gastroesophageal reflux disease (GERD) is a digestive disease which has increased in prevalence in recent years [1]. The definition of GERD is symptoms of mucosal damage produced by reflux of gastric acid across an incompetent gastroesophageal junction. It may lead to esophagitis, peptic esophageal ulcer, esophageal stricture, Barrett's esophagus, and esophageal adenocarcinoma [1, 2]. A reliable, noninvasive technique, such as barium radiography, is clinically useful for diagnosing esophagitis, but relatively insensitive than endoscopy. Invasive endoscopy remains the gold standard for diagnosing esophagitis, performed by gastroenterologists as routine practice. Previous studies have investigated noninvasive means of diagnosing esophagitis using radiopharmaceuticals (^67^Ga, ^99m^Tc-pertechnetate, ^201^Tl, ^99m^Tc-Methoxyisobutylisonitrile) [3–6]. However, poor sensitivity and image resolution problems restricted their use. Functional imaging, led by FDG-PET imaging, is likely to play an increasingly critical role in assessing inflammatory disorders [7, 8]. However, only a few previous studies have reported FDG uptake in esophagitis. In 1999, Bakheet et al. reported FDG uptake in benign esophageal diseases in patients with postradiation bacterial esophagitis, Barrett's esophagus, and gastroesophageal reflux. The patterns of FDG uptake exhibited some differences among these diseases. Bacterial esophagitis has an intense linear pattern visible in the entire esophagus. In Barrett's esophagus, uptake usually occurs in the distal one third of the esophagus. One patient with gastroesophageal reflux demonstrated mild focal lower esophageal uptake [9]. In 2005, Bural et al. reported a case of reflux esophagitis secondary to chemotherapy, in which diffuse intense FDG uptake occurred in the entire esophagus [10]. A previous report provided images of esophagitis, detected using FDG-PET, characterized by mild-to-moderate FDG uptake activity in the esophagus. The purpose of the present study was, therefore, to detect and evaluate various degrees of gastroesophageal reflux esophagitis using FDG-PET.

## 2. Materials and Methods

### 2.1. Participants

This is a retrospective study, reviewing the charts of healthy subjects for health screening examinations, who were referred from the Department of Family Medicine, China Medical University Hospital during September 2008 to December 2009. 408 subjects (169 females and 239 men, ages ranging between 25 and 86 with an average of 52.1), who underwent both FDG-PET and upper gastrointestinal endoscopy within a two-week period, were assessed for comparison based on similar clinical conditions. This study was approved by the hospital ethics committee (DMR-99-IRB-010).

The findings of the endoscopy and FDG-PET were separately evaluated by one endoscopist and two FDG-PET readers with agreement. The endoscopy findings followed the modified version of the Los Angeles classification to describe the different grades of severity of esophagitis (M and A to D) based on the extent of esophageal lesions [11, 12]. Findings and diagnoses were recorded separately on the electronic record system to create the participants' database.

### 2.2. PET Imaging

PET studies were performed using Advance NXi PET scanner (General Electric Medical Systems, Milwaukee, WI) for 40 min to 1 h after the intravenous injection of 370 MBq (10 mCi) of 18F-FDG. Before PET scanning, the serum glucose levels of all subjects were checked to ensure the readings were less than 180 mg/dL. Scanning was performed from the head to the upper thigh in 2D mode 4 min per bed position. Transmission scans were acquired with ^68^Ge rod sources for attenuation correction. Reconstruction of transmission and emission scans used ordered-subset expectation maximization. The images were reconstructed and displayed in 3D and axial, sagittal, and coronal reconstructions for interpretation. Quantitative analysis of ^18^F-FDG uptake in the esophagus region was performed and maximum standard uptake value (SUVmax) was calculated.

### 2.3. Statistical Analysis

The distribution of gastroesophageal reflux esophagitis levels was skewed to the right, therefore, natural log-transformation for gastroesophageal reflux esophagitis (ln gastroesophageal reflux esophagitis) was used to normalize the data.Comparisons of mean values of ln gastroesophageal reflux esophagitis (geometric means of gastroesophageal reflux esophagitis), according to the grades of esophagitis severity, were performed using analysis of variance and post-hoc comparison with Bonferroni correction. Results are expressed as mean ± standard deviation, and geometric means. All analyses were conducted using SAS version 9.2 (SAS Institute Inc., Cary, NC). A *P*-value less than 0.05 was considered statistically significant.

## 3. Results

Table 1 shows the demographic characteristics of normal subjects and patients with gastroesophageal reflux esophagitis. There were no statistical differences in age, gender, and body mass index (BMI) between these two groups.  Table 2 lists the comparative results of the 408 participants who underwent both FDG-PET and upper gastrointestinal endoscopy. FDG-PET findings indicated that 275 of these participants had suspected lesions of the gastroesophageal reflux esophagitis, and 47% (=128/275 × 100%) of these cases received a diagnosis of reflux esophagitis. The modified version of the Los Angeles classification indicated the relevance between FDG uptake and endoscopic findings of gastroesophageal reflux esophagitis. The positive percentages detected by FDG-PET in grade M, grade A, as well as grades B and C were 71% (=40/56 × 100%), 82.9% (=68/82 × 100%), and 100% (=20/20 × 100%), respectively.

**Figure 1:**
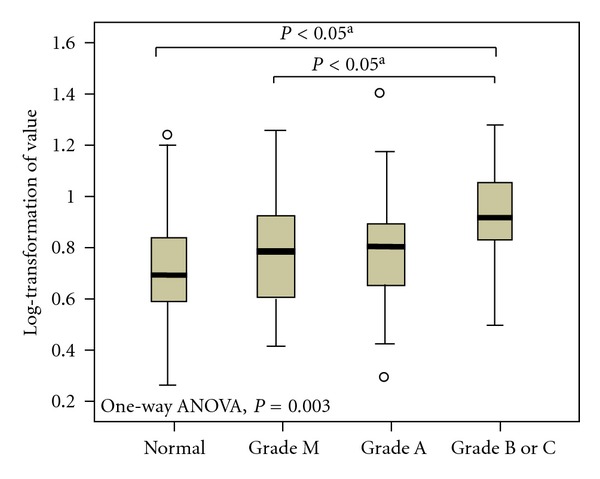
Distribution of geometric means of FDG uptake (SUVmax) and standard deviation according to the grades of severity of esophagitis. One-way ANOVA and post-hoc comparison with Bonferroni correction compared mean values of ln SUVmax (log-transformation SUVmax) among the grades of esophagitis severity.

**Figure 2:**
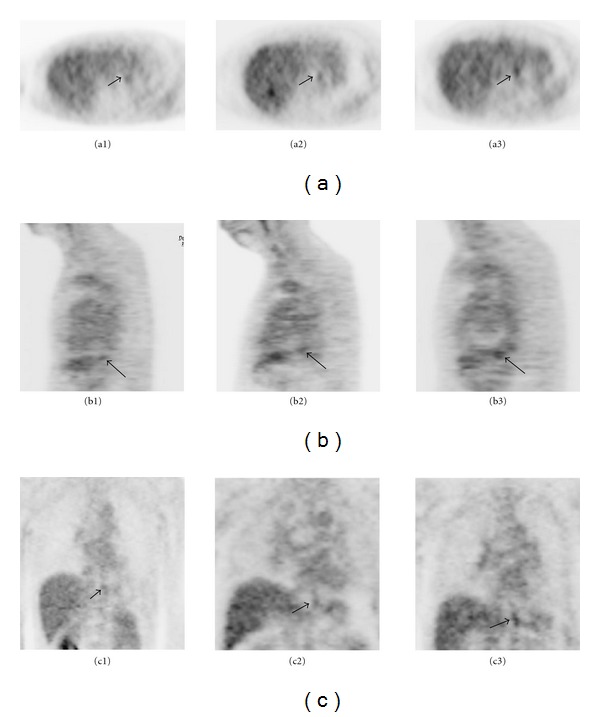
FDG-PET images showing increased FDG uptake in the distal esophagus (arrows) in the esophagitis group. (a) Transverse view, (b) sagittal view, and (c) coronal view. The maximum SUV of grade M (a1, b1, c1), grade A (a2, b2, c2), and grade B, and 3C (a3, b3, c3) was 2.3, 2.8, and 3.0, respectively.

Table 3 and Figure 1 show the SUVmax in normal subjects and subjects with gastroesophageal reflux esophagitis. The SUVmax ranged from 1.30 to 3.40 with a mean value of 2.10 and median value of 2.00 in normal subjects, and ranged from 1.30 to 4.00 with a mean value of 2.23 and median value of 2.25 in subjects with gastroesophageal reflux esophagitis. In the gastroesophageal reflux esophagitis group, the mean SUVmax for patients with modified version of the Los Angeles classification grade M, grade A, and grades B and C were 2.1, 2.2, and 2.5, respectively (Figure 2). One-way ANOVA and post-hoc comparison with Bonferroni correction identified statistical differences between the three groups (*P* = 0.003).

## 4. Discussion

GERD occurs more frequently in Europe and North America than in Asia, but its prevalence is increasing in many Asian countries. In Western countries, the prevalence ranges from 20% to 40% [13, 14] and in Asian countries, from 5% to 17%. By country, the prevalence is 12.4% in Taiwan, 7.7% in Japan, and 20% in Turkey [1, 15]. The epidemiology of GERD differs between Asian and Western populations. Erosive esophagitis and the complications of GERD, including Barrett's esophagus and adenocarcinoma, were less common than in Western countries. Erosive esophagitis, mostly grade A or B, and nonerosive reflux disease (NERD), are more common in Asian than in Western countries [16].

 The mechanism of FDG-PET uptake in esophagitis is similar to that in malignant tumors (glucose transporters transport FDG to cells and hexokinase enzyme phosphorylates FDG to FDG-6-phosphate (FDG-6-P), but it does not metabolize it). Higher FDG uptake reflects higher glycolytic rate in these processes. Prior studies have also proposed this as the mechanism of increased FDG uptake during PET examination in granulomatous diseases, skeletal infections, fever of unknown origin (FUO), vasculitis, HIV-AIDS, organ transplantation, and inflammatory bowel diseases (IBD) [17, 18].

 In the authors' experiences, the accumulation of FDG-PET in the distal esophagus is not an infrequent situation, especially in patients with gastroesophageal reflux, but few previous studies have described this phenomenon. Incidental abnormal findings in the esophagus in FDG-PET are not specific for esophagitis. Esophagitis may have various causes including reflux, infection, or physiological uptake, and abnormal FDG uptake in the esophagus may represent tumor or Barrett's esophagus. These different potential causes of abnormal PET findings may not be distinguishable by the intensities or distribution patterns of FDG uptake.

 The present study evaluated the use of noninvasive FDG-PET to detect gastroesophageal reflux esophagitis in an asymptomatic population, for health screening purposes, at a major medical center in Taiwan. Results demonstrated that noninvasive FDG-PET may be useful in the detection and characterization of the various degrees of gastroesophageal reflux esophagitis. Using invasive endoscopy as the reference standard, there was a good correlation between the severity of esophagitis detected using endoscopy and the degree of abnormal FDG uptake on PET as assessed quantitatively by SUV, or more specifically, a log transformation of the SUV.

 In this study, there were 30 subjects with less severe esophagitis; 16 grade M and 14 grade A lesions as seen on endoscopy, whose FDG-PET examinations were negative by visual interpretation, that is, uptake was not more than background activity. These cases might be false negatives due to small or less severe lesions and the limited spatial resolution of PET, or they might arise due to the performance of FDG-PET and endoscopic examinations at different times. 

In conclusion, although FDG-PET may not have perfect sensitivity, the results of this study suggest that detection of incidental abnormal findings in the distal portion of the esophagus using FDG-PET should prompt further evaluation using an anatomic imaging technique (such as PET/CT) for precise localization of the abnormal uptake, or referral to a gastroenterologist for further confirmation by invasive endoscopy. 

## Figures and Tables

**Table 1:** Demographic characteristics of the study participants.

		Age	Sex		BMI
	No	Mean ± SD	Female	Male
Normal	250	51.42 ± 9.81	116	134	24.10 ± 3.88
Esophagitis	158	53.20 ± 9.73	53	105	24.17 ± 3.81
Grade M	56	54.09 ± 11.76	28	28	23.87 ± 4.00
Grade A	82	52.18 ± 8.62	23	59	24.17 ± 3.85
Grade B and C	20	54.85 ± 7.42	2	18	25.78 ± 3.38

**Table 2:** Comparative results of the subjects who underwent both FDG-PET and upper gastrointestinal endoscopy.

	Endoscopic finding			
	Modified Los Angeles classification			
	Normal	Grade M	Grade A	Grade B & C
FDG-PET positive	147	40	68	20
FDG-PET negative	103	16	14	0

Total	250	56	82	20

**Table 3:** FDG uptake (SUVmax) in normal subjects and subjects with gastroesophageal reflux esophagitis in FDG-PET.

Group	No	Lowest	Highest	Mean ± SD	Median
Normal	147	1.30	3.40	2.10 ± 0.41	2.00
Esophagitis	128	1.30	4.00	2.23 ± 0.45	2.25
Grade M	40	1.50	3.40	2.13 ± 0.42	2.15
Grade A	68	1.30	4.00	2.21 ± 0.45	2.20
Grade B and C	20	1.60	3.40	2.48 ± 0.47	2.40
